# Who Is Our Guide, and Where Shall We Find Him?

**Published:** 1885-02

**Authors:** 


### WHO IS OUR GUIDE, AND WHERE SHALL WE FIND HIM?

Each month as my journals come to hand I read them, with Allen, Lippe, Guernsey, and Burt at my side. But so often I find myself without a guide that I am almost lost.

Having a case this last week in which the patient complained of cold heels, which sweat offensively and profusely in summer, I looked in my guides in vain for the thread. Several years ago a journal issued under the auspices of the Cleveland Hospital College gave a case like the above (except the coldness) cured with Sep. I gave it to my patient, and lo! the same day I received my *Medical Advance* (October), which had been wandering in the mail, and Dr. H. N. Guernsey gave Bary c., Graph.,

Kali c., Nit. ac., Sep., Thuja, and Sel. with offensive and profuse sweat of feet; while Lippe gives Sil. only, and Allen Sil. and Graph.

Now it seems to me that it is not an eliminated materia medica that we want, but one containing just this class of symptoms, which enables such men as Guernsey, Lippe, Kent, Bayard, etc., to master their knotty cases. A repertory and materia medica with this class of information would be an acquisition.

Take the case of Dr. Kent's, in which he cured the spasms of the face. My armamentarium is silent upon such fine discriminations. If any one in the profession can put those who are novices in possession of just this class of information it will be a star in their crown. The work accepted by Drs. Dake and Hughes may be useful to some, but I cannot see how it would be useful in such cases as those referred to.

Dr. P. P. Wells is a writer I love to read after; and yet he so frequently leaves out the key to his case—as instance, Vol. III, p. 174. He wrote me it was cured with Hep. sulph.

Gentlemen, do not forget we need clear-cut work, showing out like Dr. Kent's reports.

